# Stemness in Cancer: Stem Cells, Cancer Stem Cells, and Their Microenvironment

**DOI:** 10.1155/2017/5619472

**Published:** 2017-04-04

**Authors:** Pedro M. Aponte, Andrés Caicedo

**Affiliations:** ^1^Colegio de Ciencias Biológicas y Ambientales, Universidad San Francisco de Quito (USFQ), 170901 Quito, Ecuador; ^2^Colegio de Ciencias de la Salud, Escuela de Medicina Veterinaria, Universidad San Francisco de Quito (USFQ), 170901 Quito, Ecuador; ^3^Mito-Act Research Consortium, Quito, Ecuador; ^4^Colegio de Ciencias de la Salud, Escuela de Medicina, Universidad San Francisco de Quito (USFQ), 170901 Quito, Ecuador; ^5^Colegio de Ciencias Biológicas y Ambientales, Instituto de Microbiología, Universidad San Francisco de Quito (USFQ), 170901 Quito, Ecuador

## Abstract

Stemness combines the ability of a cell to perpetuate its lineage, to give rise to differentiated cells, and to interact with its environment to maintain a balance between quiescence, proliferation, and regeneration. While adult Stem Cells display these properties when participating in tissue homeostasis, Cancer Stem Cells (CSCs) behave as their malignant equivalents. CSCs display stemness in various circumstances, including the sustaining of cancer progression, and the interaction with their environment in search for key survival factors. As a result, CSCs can recurrently persist after therapy. In order to understand how the concept of stemness applies to cancer, this review will explore properties shared between normal and malignant Stem Cells. First, we provide an overview of properties of normal adult Stem Cells. We thereafter elaborate on how these features operate in CSCs. We then review the organization of microenvironment components, which enables CSCs hosting. We subsequently discuss Mesenchymal Stem/Stromal Cells (MSCs), which, although their stemness properties are limited, represent essential components of the Stem Cell niche and tumor microenvironment. We next provide insights of the therapeutic strategies targeting Stem Cell properties in tumors and the use of state-of-the-art techniques in future research. Increasing our knowledge of the CSCs microenvironment is key to identifying new therapeutic solutions.

## 1. Introduction 

Cancer is a major cause of death worldwide [1, 2]. While the incidence of infectious diseases has significantly declined over the last several decades, overall incidence of solid tumors and leukemia has shown to be increasing [3]. Longer average life span, accumulation of genetic mutations, and permissive microenvironment are key factors promoting cancer progression [4, 5]. Most therapies include the use of strong cytotoxic molecules to target specific unregulated factors to eventually affect cell proliferation and survival of the tumor [6]. Due to its fast replication capacity and constant mutations, cancer adapts to aggressive environments and can persist after therapeutic management. Stemness of cancer cells is a key feature for cancer progression and in many cases the source of its survival [7–12]. Understanding the development and acquisition of resistance in cancer cells may therefore provide opportunities for more effective therapies.

Stem Cells (SCs) have the capacity to self-renew and give rise to progeny capable of differentiating into diverse cell types [13]. SCs cannot survive either outside their environment or in the absence of specific factors and cytokines [14, 15]. Interestingly, the environment and/or specific stimuli can promote the emergence of new SCs, as cells in general maintain the ability to dedifferentiate and return to a primitive state of development [16–18]. Such capacities are comprised in the term stemness and correspond to cells devoid of differentiation marks [19, 20].

Malignant cells develop all aspects of stemness, fail to sustain tissue homeostasis, and, contrary to the physiological role of adult SCs, sustain the progression of cancer disease [8]. Stemness features common of SCs and cancer cells provide the building blocks for cancer maintenance and survival, from self-renewal and differentiation potential to the organization of stemness supporting microenvironments [5, 9, 21]. Thus, Cancer Stem Cells (CSCs) are a small population of cells within tumors holding stemness properties that sustain cancer progression, such as enhanced capacities for self-renewal cloning, growing, metastasizing, homing, and reproliferating. CSCs show remarkable organizing capacities as they can educate neighboring cells to provide nutrients and collaborate in the elusion from the immune system, creating an environment favorable for tumor growth. CSCs give rise to heterogeneous cell populations, often with a high plasticity potential [10, 22], high resistance to stressful factors within the tumor microenvironment (such as low oxygen or nutrient levels) or to the induction of cell death by chemotherapeutic agents [11, 23], and quiescence as a common response [12, 24].

In order to understand how we can take advantage of stemness to develop applications in the field of oncology, this review will discuss the most relevant known stemness features shared by adult SCs and CSCs in normal tissues and tumors, from the origin and progression to the outcome. As stemness involves the organization of a microenvironment that protects normal SCs (Stem Cells) niche or CSCs (the Tumor Microenvironment, TME) we will present the most common companions of cancer cells and their interactions within the TME. Among such neighbors of SCs and CSCs, Mesenchymal Stem/Stromal Cells (MSCs) are the main contributors to the maintenance of stemness, as they provide support to the niche and the TME during stress and generate an immune-privileged regulatory microenvironment [25, 26]. Therefore, we will provide insights into the particular contribution of MSCs to cancer. As cancer cells are continually readapting to conventional therapies, current research is constantly evolving to generate new approaches to effectively target their progression. Many of these therapeutic procedures show an increasing trend towards personalization. They aim to affect the hallmarks of cancer development and, in particular, the stemness elements affecting specific patients. Therefore, the current understanding of the mechanisms underlying stemness in tumors will be covered in this review, in the context of new therapies potentially targeting the organized TME.

## 2. Adult Stem Cell Characteristics

All tissues in the body organize their functions around cellular communities essentially conforming microenvironments, where SCs play a key role in the general homeostasis. Through well-regulated asymmetric cell divisions, SCs provide the progenitors that will in turn generate specialized daughter cells responsible for maintaining organ functions and replacing wear-and-tear cell losses [27]. At the same time, SCs are able to self-renew with the purpose of regulating their numbers under both physiological and abnormal conditions [28]. Adult (nonembryonic) SCs all have by definition some degree of differentiation potential. Therefore, adult SCs have the capacity to produce several differentiated lineages, a differentiation potential restricted to multipotency. Adult SCs are located in specialized microenvironments that provide support and cues that instruct them to maintain themselves and self-renew as required by local cell dynamics in specific tissues [29–32]. Although located in multiple different niches in several tissues of the body, SCs share common features such as self-renewal capacity, undifferentiated state combined with differentiating potential, long cell cycling, genome repair abilities, and microenvironmental protection by the niche itself when under attack from a wide range of insults [27]. The term stemness condenses all key properties of SCs, defined by specific patterns of gene expression or epigenetic status within the context of the tissue where they reside. In the skin, a well-characterized tissue in terms of SC activity, epidermal SCs (interfollicular keratinocyte progenitor cells) express *β*1-integrin while their progenitors do not [33]. In the small intestine SCs express specific sets of stemness determining genes [34]. The characterization of SC's specific gene product profiles can be conveniently used to track them along their cellular dynamics in specific body systems [35]. In most tissues, SCs are located at the top of hierarchical organizations collectively called Stem Cell Systems (SCSs). Thus, almost every major organ in the body has at least one SCSs. Even organs long-held as not prone to regeneration are now appearing to show SCs activity under certain conditions. For instance, the long-held dogma of global terminal differentiation in adult neurons has been strongly challenged [36].

**Figure 1:**
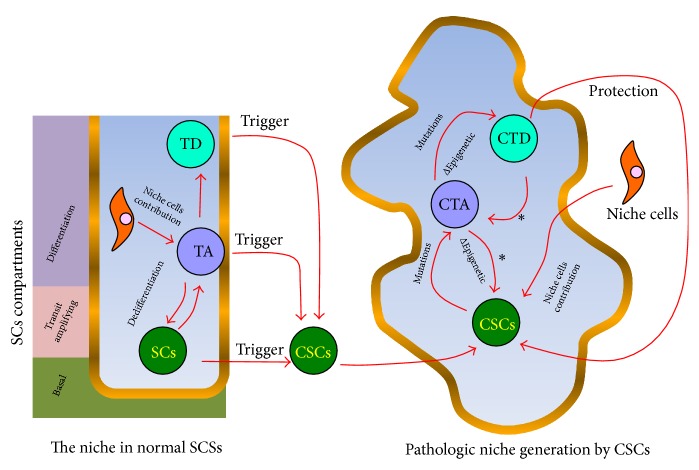
The origin of Cancer Stem Cells (CSCs) and Stem Cells (SCs) involvement in the generation of pathological cell hierarchies in tumors. In normal Stem Cell Systems, SCs located at the basal compartment generate committed progenitors (through asymmetrical divisions) which become spatially relocated to the transit-amplifying (TA) compartment. There, progenitors actively divide to produce differentiated daughter cells that carry on the normal physiology of the organ. Under physiological emergencies associated with SC loss, TA cells can dedifferentiate to reload the SC pool. Certain stressful triggers (i.e., chronic inflammation, ROS accumulation, and aging) can promote the transformation of cells in the system and generate CSCs or cancer initiating cells. CSCs remodel the niche and produce a pathological cancer microenvironment and associated hierarchy (pathological Stem Cell System) that resembles the original normal Stem Cell Systems (SCSs). The tumor is a very heterogeneous entity with cells that have accumulated mutations and epigenetic profile changes to secure CSCs survival and thriving. Features typical of SCSs such as niche support, SCs stemness, and dedifferentiation paths (*∗*) remain in the tumor environment. SCs = Stem Cell; TA = transit-amplifying progenitor; TD = terminally differentiated cell; CSC = Cancer Stem Cell; CTA = cancer transit-amplifying progenitor; CTD = cancer terminally differentiated cell.

SCSs consist of (1) basal, (2) transit-amplifying, and (3) differentiation compartments [27].  Figure 1 summarizes the structure of SCSs and some possible ways of transformation into a Tumor Stem Cell System. The basal compartment is where SCs reside. That compartment, including its immediate surroundings, corresponds to the SCs niche where other cells, Extracellular Matrix (ECM), and factors like oxygen levels and physical forces contribute to the maintenance and survival of SCs [29]. Cellular components of this niche include local elements (very often, cells of mesenchymal origin) or immune cells recruited to the site [25, 37, 38].

The direct SCs progeny or transit-amplifying cells occupy the transit-amplifying compartment. These transient-in-nature cells have a shorter cell cycle than their mother SCs and they therefore rapidly divide to produce daughter cells that “amplify” the next compartment (differentiation compartment), where terminally differentiated cells that perform normal tissue/organ functions dwell [27]. Transit-amplifying cells, also called progenitor cells (or progenitors), are morphologically similar to their SCs ancestors but show different sets of markers that define their differentiation commitment. However, under certain circumstances they dedifferentiate and contribute to the SCs pool. An example of such a dedifferentiation process occurs in the seminiferous epithelium in the testis, where spermatogenesis, the Spermatogonial Stem Cells- (SSCs-) dependent system, generates sperm [39]. In this well-characterized SCs system, SSCs divide asymmetrically to produce differentiated daughter cells that, through mitosis (transit-amplifying activity), generate clones that remain connected through intercellular bridges [40]. Any exogenous process interfering with intercellular bridge integrity (i.e., irradiation) will produce individual single undifferentiated SCs, reversing the differentiation process back to the SCs level [41, 42]. These initial observations were more recently corroborated through in vivo experiments and functional tests for SCs capacity [43]. SCSs are thus tightly regulated cellular hierarchies where SCs activity, modulated by the niche, follows a proper balance between self-renewal and differentiation in order to maintain normal organ activity.

## 3. Analogous Features of CSCs and Adult SCs 

There is growing evidence that cancer disease follows SCSs organization where cancer cells or CSCs generate a comparable hierarchical structure within tumors (Table 1). The Theory of CSCs is a modern derivation of the Embryonic Rest Theory of cancer. This Theory states that vestiges of embryonic tissue would remain in adult postnatal organs while holding the capacity to pathologically unbalance the surrounding tissues (Field Theory), therefore leading to a situation in which the remnant embryonic tissues start proliferating into a tumor mass whose cells are similar to the embryonic cells of origin [44]. The existence of teratoma tumors supports the Embryonic Rest Theory, since embryonic Primordial Germ Cells (PGCs) give rise to this kind of tumor in adult-age locations which are spatially associated with their prenatal migration path into the genital ridge, where either testes or ovaries eventually develop [45].

Several other models proposed to explain the origin of cancer cells (chemical carcinogenesis, infections, mutations, and epigenetic changes) are likely to involve dedifferentiated cells with SCs properties. Consequently, many cancers could arise from the maturation arrest of adult SCs in different tissues [44, 46]. The origins of CSCs are traceable with techniques previously used to uncover unipotent or multipotent SCSs under normal physiological conditions [47]. Blanpain (2013) [33] traced tumor initiation back to SCs in several known SCSs through the use of recombinant Cre-Loxp technologies. It is now possible to conditionally express oncogenes or delete tumor suppressor genes through the targeted activation of Cre recombinase expression in solid tumors in order to trace their cellular origin to one precise cell [33]. In some tumors in which progenitors appeared to be the initiating tumor cells, a dedifferentiation process generating primitive CSC that feed the cellular hierarchy has been found [22, 46].

Currently, a fundamental question in cancer biology is whether there is order within the chaos inside cancer masses. Tumors in and of themselves are very complex biological entities; they are heterogeneous aggregations of cells disorganized to the point of chaos. Within a disorder that seems to prevail, remnants of an orderly arrangement of normal tissues become apparent after careful analysis. Thus, knowledge about the origin of cancer cells becomes crucial to understand cell heterogeneity in cancer. In a Stochastic Model of Cancer Cell Dynamics, mutations giving rise to cells with unrestricted division capacities occur at random. Transformed clones suffer successive mutations along their descendant lines in branching patterns [10]. The high mutation rates found in tumors increase the likelihood of developing clones adapted to the tremendous selection pressures present at the tumor site (i.e., local chemotherapeutic agents, radiation, ROS, and immune attack).

A more recent model of cancer, the CSCs Model, covers issues not completely explained by the Stochastic Theory, such as tumor recurrence after treatment. The CSCs Model states that surviving, transformed subclones that form part of tumors have SCs properties that allow them to drive tumor progression. The CSCs Model is unidirectional in that SCs-like cells may generate progenitor daughter cells (transit-amplifying cells) that in turn divide to produce differentiated (nontumorigenic) cells. Cellular heterogeneity within tumors depends on factors including the already mentioned branching mutation patterns and on cues from the TME. Thus, the TME can contribute to cell transformation (Figure 1).

**Figure 2:**
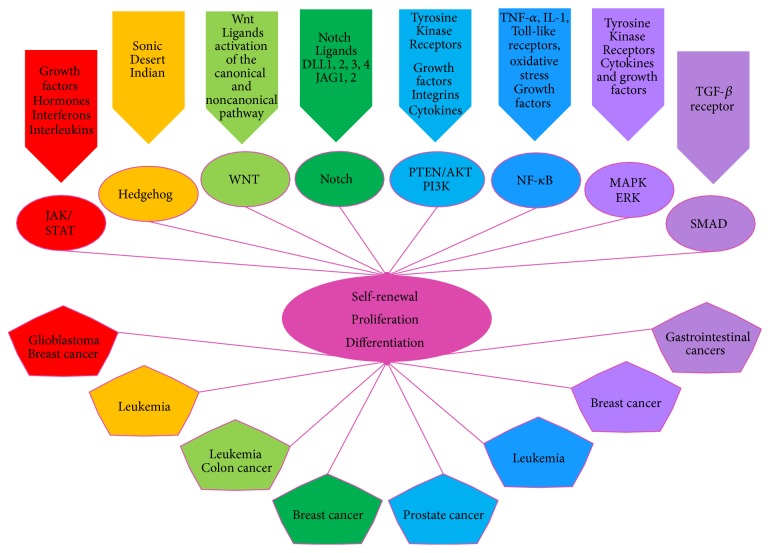
Common signaling pathways between Stem Cells (SCs) and Cancer Stem Cells (CSCs) [48]. CSCs share common signaling pathways, like the JAK/STAT, Hedgehog, Wnt, Notch, PTEN/AKT/P13K, NF-*κ*B, MAPK/ERK, and SMAD. These SCs mechanisms are altered in CSCs and are characteristic of the cancer types mentioned. The JAK/STAT pathway (Janus kinase/signal transducer and activator of transcription) is mainly involved in glioblastoma development and breast CSCs [49–52]. The Hedgehog pathways have effects on the patterning of the embryo but play a crucial role in the induction of myelogenous leukemia. Blocking of the Hedgehog pathway decreases the quantity of CSCs in leukemia, then representing an important target for cancer therapy [53]. The Wnt pathway is an important regulator of SCs and CSCs regarding self-renewal, being perturbed in colon cancer and leukemia [54–56]. The Notch pathway is involved in the development of breast tissue as a regulator of cell fate and differentiation. An excess in the activation of Notch could determine the aggressiveness of breast cancer [55, 57–59]. The phosphatase and tensin homolog (PTEN)/protein kinase B (PKB or AKT)/phosphatidylinositide 3-kinase (P13K) signaling is a key regulator of self-renewal and maintenance of SCs and CSCs with an important role in the emergence of CSCs in prostate cancer [51, 60]. The NF-*κ*B pathway is crucial for leukemic cells survival and its inhibition affects CSCs development in breast cancer [61]. It has been seen that the increase of neural stem cell (NSC) proliferation is caused by the activation of NF-*κ*B, through the TNF-*α* signal transduction pathway, but its aberrant regulation could lead to CSCs development in glioblastomas [62, 63]. Blocking the mitogen-activated protein kinase (MAPK)/extracellular signal-regulated kinase (ERK) results in the growth inhibition of breast cancer and the emergence of CSCs, sensitizing cancer cells to chemotherapy [64–66]. Gastrointestinal SCs can be perturbed, changing their plasticity and differentiation potential by generating an aberrant response to TGF-*β* affecting the SMAD pathway and generating CSCs [67]. The hepatocellular carcinoma is an aggressive form of cancer in which the TGF-*β*, Notch, and Wnt are deregulated, also having consequences in the SMAD proteins and changing SCs renewal, differentiation, and survival patterns [68, 69]. In adult and CSCs systems all the mentioned pathways are common and conserved in the control of SCs renewal, proliferation, and differentiation.

Similarly, as in normal SCSs, there is growing evidence that indicates progenitor cell pools within tumors revert back to CSCs by several means such as Epithelial Mesenchymal Transition (EMT) [70, 94]. EMT and plasticity are related processes that are associated with cancer progression. Multiple potential cell fate paths among the pool of progenitors and CSCs add a high degree of complexity to the cell dynamics of the cancer model. Plasticity has been termed “dynamic stemness” in this context [10]. Thus, plasticity, usually mediated by microenvironmental signals, is another very important mean for gaining excessive SCs self-renewal properties in tumor environments. Many of the mutations found in tumors are involved in the activation of self-renewal pathways in one way or another [70, 94]. In cancer, cells' multiple self-renewal pathways can not only be enhanced but also become continuously activated in ways that are only subtly different from the self-renewal pathways of normal tissues [72]. This self-renewal program activation forms an integral part of CSCs stemness, actively promoting tumor progression and metastasis by generating a high cell turnover and production of progenitors. Thus, a pathogenic self-renewal over differentiation balance in tumors further aggravates the process of mutation accumulation (Figure 2).

Another cause of cellular heterogeneity in tumors studied in recent times is epigenetics. Tumor complexity and plasticity can hide a hierarchical organization within the TME in part because of altered epigenetic profiles that may adopt mutation phenotypes. Deoxyribonucleic Acid (DNA) methylation changes and chromatin remodeling have been detected in many types of cancer [46, 95]. Overall, DNA methylation is enhanced, causing many differentiation genes to shut down [96]. The Polycomb group of proteins is one of the epigenetic regulators in cancer and SCs. The Polycomb Repressive Complexes (PRCs), active in binding to the CpG-rich promoters of genes controlling development and differentiation in embryonic SCs, are involved in the transcriptional repression [96]. The inhibition of one of such complex, PRC2, is being explored as a new cancer-treating therapy because its deletion has been shown to inhibit tumor progression [97]. However, since different types of cancer have different genetic and epigenetics profiles, the ablation of PRC2 could also cause cancer cells to become more aggressive by reinforcing their phenotype [98]. When tumor suppressor or differentiation genes are altered through the abnormal activation of epigenetic mechanisms, more resources are added to the toolkit that allows tumor survival and evolution within the TME [46].

## 4. Development of the CSCs Population in the TME

As previously mentioned, cancer is the product of cells deviating from normal tissue regulation mechanisms, due to the accumulation of oncogenic mutations with survival advantages over other cells [99]. During carcinogenesis any cell type is prone to malignant transformation depending on the degree of accumulation of nononcogenic or oncogenic mutations [21, 100]. Normal SCs, progenitor cells, or differentiated cells can escape regulation and become a CSCs, a cancer progenitor cell, or a poorly regulated differentiated cell [101] (Figure 1). Tumor Initiating Cells (TICs) can be traced back to CSCs, but CSCs are not always the cell of origin of the fittest clones in cancer [31]. The great variety of mutations within a tumor give rise to the many different phenotypes and variations in plasticity properties [102]. A poorly regulated cell can transform into a cancer progenitor cell leading to an expression profile similar to that of SCs, but there is no direct evidence that a poorly regulated cell could eventually become CSCs (even if it passes through all the transformation stages) [23, 103, 104]. Hematopoietic bone marrow represents a good example of how cancer can arise from the accumulation of mutations due to high tissue turnover [105]. Hematopoietic Stem Cells (HSCs) constantly accumulate DNA damage due to physiological stress produced by infections or persistent blood loss, contributing to age-related tissue degeneration and malignant transformation [100, 104]. SCs' constant transition out of dormancy and the subsequent continuous proliferation of their progeny can lead to the development of CSCs [106]. In several types of human leukemia, mutant HSCs prevail over normal HSCs [106]. This unbalance has been associated with mutations that increase the activity of Ras signaling, which in turn favors the presence of cancerous HSC clones in the niche [106].

The organization of the tumor can be mediated by CSCs cytokines which induce changes in the cells that make up the TME and thus generate a cancer niche [107]. The presence and proliferation of CSCs or TICs stimulate the organization of the TME, providing the tumor with more heterogeneity and fueling its aggressiveness [108]. Very actively self-renewing and Long-Term TICs (LT-TICs) have been observed in colon cancer from the first stages of development. Additionally, it has been observed that they are able to maintain tumor progression in murine xenotransplants [109]. Tumor Transient Amplifying Cells (T-TACs) with less self-renewal capacity/metastatic potential and Delayed-Contributing TICs (DC-TICs) do not become activated in primary tumors but contribute to the advance of the disease only after transplantation [110]. These cells with different self-renewal capacities and nontumorigenic progeny generate the necessary genetic heterogeneity within tumors that allows for the existence of clones that withstand chemotherapy [81]. Other cells, like MSCs and macrophages, can be attracted to the tumor site and fuel the tumor with prosurvival factors that promote the cancer development [26]. Through this process, multipotent MSCs cells support the stemness of tumor tissues in the same way that they would function during normal SCSs homeostasis. MSCs are able to react, for example, to Interleukin-1 (IL-1) secreted by cancer cells and produce Prostaglandin E_2_ (PGE_2_) [111]. In normal SCs niches, PGE_2_ regulates the amplification of multipotent progenitors and therefore represents a key factor in the homeostasis of the HSCs during stress [112, 113]. In the HSCs niche, MSCs contribute to the maintenance of normal HSCs as well as the organization of the CSCs microenvironment [114]. Thus, MSCs are key players in the maintenance of the stemness in both adult SCs and CSCs microenvironments, helping them to survive physiological stress and therapies [83, 115, 116].

Inside the TME, distinct cancer clones struggle to endure environmental pressures (e.g., lack of nutrients, oxygen, and immune surveillance). The hierarchical organization of cells created by selective processes benefits the establishment and survival of CSCs [23, 117]. Cells surrounding the CSCs niche cooperate to create a microenvironment that shares several common features with normal SCs niches (Table 2). Both types of SCs niches, normal and tumoral, set the stage for complex interactions with hematopoietic cells, endothelial cells, fibroblasts, MSCs, soluble signaling elements, and the ECM to create an immune-privileged environment, [118]. As cancer cells approach a SCs phenotype they become more resistant to chemotherapy and assume the top position of the TME hierarchy. The analysis of high burden (advanced-stage metastatic disease) versus low burden (early-stage metastatic disease) patient-derived triple-negative (Estrogen Receptor Negative, ER^−^, Progesterone Receptor Negative, PR^−^, and Human Epidermal Growth Factor Receptor-2 Negative, HER2^−^) Breast Cancer Cells (BCCs) showed that low burden metastatic cells are more similar to SCs, expressing genes like* Cyclin Dependent Kinase Inhibitor 1B (CDKN1B), serine/threonine-protein kinase Chk1 (CHEK1)*,* Transforming Growth Factor Beta Receptor 3 (TGFβR3),* and* Transforming Growth Factor Beta Receptor 2 (TGFβR2)* and a quiescent phenotype with the capacity to initiate tumors when xenografted in mice [119].

## 5. Comparison between the Tumor Microenvironment and the Stem Cell Niche

Both the SCs niche and the TME are rich and complex environments that combine cellular and noncellular components to sustain stemness [120]. As mentioned before and similar to the normal SCs niches, the TME is made of a mix of cells (i.e., hematopoietic cells, endothelial cells, fibroblasts, and MSCs) together with noncellular components (i.e., nutrients, growth factors) that all together sustain the survival of the CSCs and cancer progression [23, 84, 121, 122]. The ECM is an important noncellular component of both SCs niches and the TME, playing different roles in each of them. The TME is a three-dimensional network mainly composed of collagens, glycoproteins, and proteoglycans, elastin, fibronectin, laminins, and other structural molecules [123]. SCs depend on the ECM architecture as a scaffold to grow and differentiate, but instability and stiffness of the ECM in the TME promote cancer development [124, 125]. Matrix-degrading enzymes secreted by normal and cancer cells can remodel the ECM. In the first case, the degradation of the normal ECM is key for tissue growth and development while in cancer it constitutes one of the first metastatic steps [125–127]. Cancers cells produce large quantities of metalloproteinases, enzymes that degrade the ECM, thereby contributing to the instability of tissue architecture and promoting invasion and tumor angiogenesis [88, 128].

TME cells of hematopoietic origin belong to two groups: (a) those coming from lymphoid lineages and (b) those from myeloid lineages. T-cells, B-cells, and NK-cells (Natural Killer Cells) are able to substantially inhibit tumor progression but in time they can be educated by the TME to help cancer cells survive and escape from immune surveillance [84]. CD4+ T helper and CD8+ Cytotoxic T Lymphocytes (CTL) are part of the main elements of the tumor microenvironment. Th1 Lymphocytes produce Interferon Gamma (IFN-*γ*), Tumor Necrosis Factor Alpha (TNF-*α*), and Interleukin-2 (IL-2), which are all essential for tumor rejection. Th1 can collaborate with Th17 to produce IFN-*γ* and Interleukin-17 (IL-17) which in turn recruit antitumor CTLs [86]. In normal SCSs and TMEs, MSCs interact with normal adult SCs and CSCs and induce them to secrete immunoregulatory cytokines such as Interleukin-10 (IL-10) and TGF-*β*, which force CD4+ T-cells to become anti-inflammatory [129–131]. Moreover, Th2 cells' interaction with the TME and particularly with MSCs inhibits immune rejection of the tumor (through the production of Interleukin-4, IL-4, Interleukin-5, IL-5, and Interleukin-13, IL-13) and promotes the presence of immunosuppressive type 2 macrophages [132–135]. Overall, MSCs present in the SCs niche and in the TME exert their immune regulatory profile, a feature common in both microenvironments [136–138].

Emerging only from the TME, fibroblasts modified through crosstalk with cancer cells become Cancer Associated Fibroblasts (CAFs) which provide growth factors, chemokines, and ECM-modifying metalloproteases and promote local tumor invasion [121]. CAFs have a spindle shape, express *α*-smooth muscle actin (*α*-SMA), and lose their normal cytokine expression profile, thereby attaining superior migratory, proliferative, and phagocytic capacities [139]. Additionally, cancer cells secrete factors that suppress the antitumoral control of the stroma. For instance, it has been observed that melanoma cancer cells secrete Platelet-Derived Growth Factor-BB (PDGF-BB) and TGF-*β*. These factors promote the transformation of fibroblasts, inducing them to express low levels of Pigment Epithelium-Derived Factor (PEDF), which in turn has been demonstrated to have anticancer properties [140]. Furthermore, it has been shown that a CAFs conditioned medium, cultured with prostate cancer cells, promotes their survival in the presence of gemcitabine, a potent chemotherapeutic agent, or after radiotherapy [139, 141].

Myofibroblasts and adipocytes, also part of the cellular stromal/endothelial cell population, are responsible for creating a tumor-permissive niche [83, 86]. Myofibroblasts, which may be present in prostate cancer, can also be educated by cytokines from cancer cells to be protumorigenic, similar to CAFs [142]. Pericytes, cells with properties similar to MSCs, are able to support tumor progression and chemotherapy survival after contact with cancer cells [83, 143, 144].

As previously mentioned, a common feature of both SCs niche and the TME is the presence of MSCs [26, 136] which are key players in cancer survival and the organization of the TME [145]. Therefore, the dialogue between MSCs and cancer cells is important to understanding other aspects of stemness in tumors. This is true in terms of the biology of CSCs, but also in terms of the participation of multipotent cells like MSCs that reside in the microenvironment or are attracted to it to promote tumor progression [146–148].

## 6. MSCs and Cancer Cells

MSCs were first identified about half a century ago when they were isolated from bone marrow and identified by their (1) capacity to adhere to plastic surfaces, (2) high potential to proliferate, (3) capacity for osteogenic, adipogenic, or chondrogenic differentiation, (4) cell surface markers such as CD105, CD73, and CD90, and (5) lack of CD45, CD34, and CD14, among others [149–151]. Although MSCs are named stem/stromal cells, they show limited stemness features which are highly variable depending on their origin, donor age, proliferation limitations, and time of isolation, even when in the presence of specific growth factors during their maintenance in vitro [152–154]. Stemness properties and the lack of a comprehensive classification of MSCs generate great controversy as they are not by definition SCs, but yet show multipotent properties [155, 156]. Even though more research is required to understand the stemness properties of MSCs, their role in providing support not only to SCs in normal niches but also to pathologic CSCs is well-documented [122, 136]. The relationship between MSCs and the TME of CSCs is essential to maintain Cancer Stemness. Current evidence indicates that MSCs and cancer cells establish a complex partnership with strong implications for tumor progression and resistance to therapy. Normally, MSCs become physiologically attracted to sites of inflammation, where they demonstrate immunomodulatory capacities while helping tissues to heal. The TME constitutes a unique site of inflammation where MSCs are able to home. Thus, TME hijacks MSCs and integrates them into the functioning of the cancer stroma in order to stimulate tumor growth and induce angiogenesis, immune evasion, and resistance to chemotherapy [145]. Cancer cells interact with MSCs, thereby leading to changes in MSCs' phenotype and inducing them to adopt features of CAFs such as the expression of*α-SMA, FSP1 (Fibroblast-Specific Protein),* or* FAP (Fibroblast-Activated Protein)* [157] or, depending on the cancer type, MSCs can further differentiate [114]. As an example, MSCs undergo osteoblastogenesis due to the secretion of Fibroblast Growth Factor 9 (FGF9) by bone metastatic prostate cancer 3 (PC-3) carcinoma cells, which results in the osteopetrotic phenotype of MSCs in prostate cancer [158, 159].

MSCs are able to increase cancer cell proliferation/survival and induce tumor metastasis [160–164]. They can also promote tissue disorganization and the EMT for Michigan Cancer Foundation-7 (MCF-7) breast carcinoma cells through cell-to-cell interactions and the secretion of paracrine factors such as TGF-*β* [165]. The rapid proliferation rate of cancer cells predisposes them to having an increased sensitivity to endogenous sources of DNA damage such as Reactive Oxygen Species (ROS), which negatively affect their survival. In such cases, MSCs reduce intracellular ROS in cancer cells in organs such as the lung through the secretion of substances that uncouple oxidative phosphorylation and direct metabolism towards glycolysis, like stanniocalcin-1 (STC1) [166]. Furthermore, MSCs contribute to cancer cells' resistance to therapeutic treatments [167]. This finding represented a major breakthrough with important clinical consequences, as cancer resistance to therapy is one of the major flaws of current cancer treatments. The role of MSCs in contributing to cancer treatment resistance has been demonstrated in Chronic Myeloid Leukemia (CML) cells [168]. An important mechanism of this process is the secretion of interleukins with dual roles in physiological conditions and cancer disease. For instance, Interleukin-7 (IL-7) plays an important role in the regulation of normal precursor T cell and B cell development. However, recent evidence shows that IL-7 promotes DNA synthesis in leukemia cells. Zhang and colleagues [169] identified a source of IL-7 in MSCs that secretes high levels of this cytokine to protect leukemic cells against apoptosis induced by Imatinib or Gleevec, a potent tyrosine kinase inhibitor. Of particular interest was another report showing that fatty acids produced by MSCs help cancer cells to survive after platinum-based chemotherapy (Cisplatin) [170]. Two unique fatty acids secreted by activated MSCs, 12-Oxo-5,8,10-Heptadecatrienoic Acid (KHT) and Hexadeca-4,7,10,13-Tetraenoic Acid, were shown to be responsible for the cancer cells' acquired resistance to Cisplatin treatment. Indeed, blocking the MSC release of these PIFAs (Platinum-Induced Polyunsaturated Fatty Acids), by targeting thromboxane synthase or cyclooxygenase-1, restored the sensitivity of Lewis lung carcinoma cells to chemotherapy in vivo [170].

The immunosuppressive properties of MSCs and their ability to attract immune cells are important partners in the promotion of tumor progression. MSCs are able to effectively inhibit the proliferation of T-cells, B-cells, NK, and dendritic cells because of their production and secretion of molecules such as TGF-*β*, PGE2, and indoleamine-pyrrole 2,3-dioxygenase (IDO) [171, 172]. Furthermore, Tumor Necrosis Factor Alfa (TNF-*α*) activates MSCs to secrete chemokine (C-C motif) ligand 5 (CCL5), C-C chemokine receptor type 2 (CCR2), and the interleukin 8 receptor, beta** (**CXCR2) ligands that in turn recruit CXCR2+ neutrophils into the tumor. The interactions between CXCR2+ neutrophils and cancer cells enhance the expression of their metastatic genes, thus activating this cancer-spreading mechanism [167]. MSCs' contribution to cancer survival is very similar to their role in tissue regeneration and maintenance of normal SCs niches [136, 173]. Among other cancer survival strategies, the TME promotes the generation of CSCs which in turn can actively educate their surroundings through interactions with other SCs (like MSCs) to guarantee self-renewal states and to give rise to the subsequent production of aggressive cells [169, 174]. Thus, MSCs' stemness-related properties, plasticity, and the ability to sustain tissue repair should be further studied in order to effectively target their cancer-supporting pathways in the TME.

## 7. Pharmacological Targeting of the Microenvironment and Cancer Stem Cells

We long ago stopped conceiving of cancer as a homogenous population of cells with a broken connection to body homeostasis. We have seen through this review that cancer is far from being a chaotic system and rather shows an organized structure, independent progression dynamics, and tremendous adaptability to environmental pressure, all factors that make full clinical remission difficult to achieve. The many tumor-promoting properties that CSCs show during cancer development establish them as pivotal as therapeutic targets in oncology. CSCs develop DNA repairing mechanisms more rapidly than their normal neighbors develop and display prosurvival factors that inhibit induced apoptotic cell death induced by chemotherapeutic agents [93]. Moreover, CSCs maintain an undifferentiated state that arms them with the ability and plasticity to survive environmental stress [93]. One way to achieve this is through Multidrug Resistance (MDR) pumps that CSCs use to extrude amphiphilic chemotherapeutic compounds like Taxanes and Anthracyclines [175]. Quiescence, another SC property key for cancer survival, allows the tumor to survive chemotherapy designed to target rapidly dividing cells [24].

Tumors tend to increase their overall volumes during their growth phase, which restricts proper vascularization and causes their centers to have low oxygen concentrations. Hypoxia in the TME promotes the generation and survival of CSCs [176]. Hypoxia activates the secretion of the Hypoxia-Inducible Factors- (HIF-) 1*α* and HIF-2*α* that can activate the expression of AlkB Homolog 5 (ALKBH5), an m^6^A demethylase enzyme reported to increase* NANOG* demethylation, thereby facilitating NANOG production. NANOG is a potent inducer of pluripotency, which contributes to the generation of CSCs [176]. The production of Carbonic Anhydrase IX (CAIX), another cancer expressed protein, is induced by hypoxia. CAIX regulates cellular pH while simultaneously promoting cancer cell survival and invasion [177]. Blocking the downstream activation of proteins induced by hypoxia (like ALKBH5, CAIX, and NANOG) has been shown to inhibit CSC expansion in the tumor site, decreasing the probabilities for tumor relapse after therapy [169, 174, 177].

The understanding of these mechanisms and the way in which SC properties in CSCs evolved in tumors in response to therapy is inspiring new effective strategies in combination with classical approaches. In an interesting recent work, Bartosh and colleagues [178] observed that BCCs internalized and degraded MSCs. In 3D coculture systems, MSCs surround BCCs, promote the formation of cancer spheroids, and then become phagocytosed by BCCs in a process mediated by Rho kinases. The engulfing of MSCs by BCCs promotes dormancy and the activation of prosurvival factors in the tumor, which is indeed a characteristic of CSCs [178]. The internalization of MSCs or its exosomes (vesicles between 40 nm and 100 nm) promotes quiescence in BBCs, favoring dormancy and relapse after the application of therapies targeting rapidly cycling cells [179].

Learning how to interrupt the interaction of MSCs with cancer cells and with the TME signaling will dramatically improve the efficiency of current chemotherapeutic options. For instance, Regorafenib or Stivarga, apart from being an oncogenic multikinase inhibitor, is also potent repressor of MSCs expression of* vascular endothelial growth factor receptors 1–3 (VEGFR1–3), Receptor Tyrosine Kinase (TIE2), PDGFR-β*, and* Fibroblast Growth Factor Receptor 1 (FGFR1)*. Thus, the use of Regorafenib suppresses the influence of MSCs over the TME, inhibiting cancer progression [180]. Platinum-based chemotherapy drugs inhibit DNA repair and synthesis in proliferative cells, making them one of the most frequently used therapeutic options to treat aggressive tumors such as those that appear in ovarian cancer [181]. Despite the fact that platinum-based drugs have achieved clinical remission with an absence of cancer disease signs or symptoms, more than half of the treated patients suffered a relapse and showed resistance to the therapy [57]. The presence of chemoresistant CSCs is the main cause of therapeutic failure. Several signaling pathways (Figure 2), such as Notch, Wnt/*β*-Catenin, and Hedgehog, play important roles in the maintenance of somatic SCs and have also been involved in CSCs' self-renewal, proliferation, and survival in the face of DNA damaging agents [182]. The successful targeting of the Notch3 pathway in mouse models affects the presence and survival of CSCs in ovarian and breast cancers, increasing the cancer sensitivity to platinum-based therapies like Cisplatin, opening new strategies to counteract the relapse of the disease [57, 93, 182].

Adenosine triphosphate- (ATP-) Binding Cassette Transporters (ABC) play an important role in cell survival as they are able to pump out toxic compounds across the cell membrane [175]. The use of CDy1 dye represents a fast and simple method to stain live SCs. Hawley and colleagues [183] reported that Multiple Myeloma (MM) cells positive for the CDy1 dye, with a SC-like gene expression signature, have an increased expression of the P-glycoprotein, a member of the ABC superfamily. The MM CDy1+ cells were resistant to the proteasome inhibitor carfilzomib, which is used in combination with other drugs like lenalidomide and dexamethasone (KRd treatment) [183, 184]. KRd treatment has been shown to promote progression-free survival but did not change a poor prognosis in patients with relapsed MM [184]. The expression of P-glycoprotein is linked to the Hedgehog pathway and it has been observed that new therapeutic drugs, such as vismodegib, show promise in sensitizing MM cells to other therapeutic drugs [183, 185].

Metastasis has been associated with CSCs that migrate from the tumor site and establish themselves in a new niche where they give rise to differentiated cancer cells. It has been shown by single cell analysis that early-stage metastatic cells possess a SCs-like expression pattern [119, 186]. These metastatic CSCs are able to resist chemotherapy through quiescence and a SC program for survival, one example of which is the Leukemia Stem Cells (LSC) in Acute Myeloid Leukemia (AML) which, as well as HSC, have mutual capacities for self-renewal and quiescence [187]. miR-126 is able to control self-renewal and quiescence in both HSCs and LSCs by the activity of P13K/AKT/MTOR, but miR-126 shows opposite outcomes. The overexpression of miR-126 in Leukemic cells enhances self-renewal and quiescence while its knockdown in normal HSCs initiates the same process. These characteristics and its knockdown of miR-126 make it a promising candidate for therapy [187]. Having the knowledge of how CSC pathways are regulated in relation to cell differentiation, renewal, and quiescence opens the possibility of targeting CSCs specific pathways without affecting normal cells [188], (Figure 2).

The continuous treatment of cancer with chemotherapeutic agents that target a single specific cell mechanism tends to facilitate the generation of resistance or in the best of cases the increase of progression-free survival, but not the cure of the disease. It is becoming more clear that targeting the SC properties employed by CSCs to self-renew, generate plasticity, survive toxicity, and/or disrupt the communication between cancer and its microenvironment could have great impact on patient remission [189]. New pharmacological combinations of compounds already available on the market to target diseases other than cancer could have a significant impact on hindering tumor progression. For instance, the use of metformin, commonly used to treat type II diabetes, in combination with 5-fluorouracil, epirubicin, and cyclophosphamide (FEC), greatly affects CSCs' ATP production, thereby impairing the cell repair mechanisms of DNA damage induced by FEC [190].

## 8. Future Perspectives in Research

The CSC field has rapidly grown in the last 20 years, generating from around 2,500 publications/year in the early 2000s to more than 5,000 in 2015 alone. This rapid progression has provided a much better understanding of SC biology [191]. Specific topics, such as the behavior of SCs in adult tissues and their mechanisms of activation, capacity for long-term self-renewal, and differentiation were key to comprehend the cellular heterogeneity of the TME, the presence of CSCs, and cancer resistance to therapy [100, 192]. From now on, the use of techniques like lineage tracing, single cell analysis, and organoid culture alone or combined will represent important tools to gain new insights into the complexity of CSCs biology and eventually to test new pharmacological compounds to target Cancer Stemness.

Cancer cells behave oddly within tumors and even cancer cell lines have shown to be heterogeneous in their proliferative potential in culture [192, 193]; this fact represents a challenge in terms of knowing the exact cellular origin of cancer. Lineage tracing using Cre-dependent marker systems, for example, takes advantage of a reporter gene to track the destiny of a cell or lineage of cells. SCs, CSCs, and other cell progeny occupying the niche are traceable with techniques that allow a better understanding of the factors activating their proliferation, differentiation, or quiescence. Lineage tracing is a fundamental tool to observe how modifications of cytokine response and their downstream signaling cascades affect cells individually, providing them with enhanced survival capacities or not. Factors such as TGF-*β* and mutations of its receptor induce changes in intestinal SCs that could lead to carcinogenesis. By using lineage tracing, Liskay and his team [194] observed that the transformation of TGF*β*R2 or its loss increased intestinal SC survival but altered their proliferation, suggesting that TGF-*β* response and sensitivity are determining factors in the sequence of events that lead to tissue transformation and cancer [194]. With the same approach, Corey and colleagues [195] demonstrated that tumor endothelial cells, while helping in the organization of blood vessels derived from a common precursor that tends to disappear, give rise to different subclones as the tumor evolves [195]. Untangling the dynamics of CSC behavior in tumors by lineage tracing will definitely open ways for a better understanding of cell transformation that usually leads to aggressive types of cancers, with the condition that similar pathways are shared by different tumors and that these mechanisms can be therapeutically targeted.

Single cell analysis by RNA-seq provides information about how a precise gene signature in SCs or CSCs determines their potential to resist stress, quiescence, proliferation, and differentiation [196, 197]. It has been hypothesized that metastasis is produced by tumor cells with unique SC properties. Using single cells analysis, Lawson and colleagues [119] observed that rare cancer cells with a SC-like gene expression profile (overexpression of* CDKN1B, CHEK1, TGFBR3,* and* TGFβ-2*) are more efficient in metastasizing and homing to other distant tissues [119]. Besides the genetic signature of a cell, the influence of epigenetic profiles on the generation of somatic mutations that lead to carcinogenesis is an important question in the understanding of cancer progression that will definitely require more research. Sunyaev and his team showed in 2015 [198] by comparing cell-type-epigenomic characteristics and mutations between diverse tumor cells that chromatin accessibility and replication timing are better predictors of their capacity to generate more mutations than the mutation signature itself. Interestingly, Sunyaev's group determined that the original cancer cell could be identified its epigenetic profile and by the distribution of mutations on its genome [198]. The identification of the cancer cell of origin by techniques like single cell analysis and lineage tracing will allow us to more clearly elucidate the dynamic of the TME heterogeneity, the presence of cancer cells progenitors, and predictions of the response of tumor cells to treatments [196].

Techniques involving mimicking the 3D cell-to-cell interactions and contact with the matrix are crucial to gaining insights into many aspects of cancer development and the generation of the CSC niche. Cells grown in 3D matrices have different gene signatures and show a better capacity to resist chemotherapeutic agents [199, 200]. The development of 3D organoid culture systems will help to understand how cell hierarchies emerge from original CSCs. Most 2D coculture systems fail to reproduce the conditions for cell-matrix interactions which are essential for processes like hypoxia generation (crucial for the CSCs phenotype), induction of the EMT, and metastasis [91, 201, 202]. BCCs interacting with fibroblasts in 3D show an enhanced invasion and secretion of metalloproteinase- (MMP-) 2 and survival cytokines [203]. Moreover, 3D culture models have been instrumental for the successful maintenance of cells that would otherwise die in 2D, such as glioblastoma cells. Interestingly, Hubert and colleagues [91] showed that organoids established from different regions of tumors from glioblastoma patients developed a fast proliferative region, a hypoxic core composed by non-stem senescent cells and quiescent CSCs. In addition, non-stem cancer cells were sensitive to radiotherapy while CSCs in the core were radio-resistant [91].

In summary, 2D cancer models and monoculture in vitro are not sufficient to address the way in which CSC niche organize and how CSCs persist after therapy. The development of 3D culture systems is fundamental to the study of the inherent heterogeneity of tumors, the details of cancer origin, and, with the use of lineage tracing and single cell analysis, how the niche evolves. Understanding which factors stimulate the persistence and division of CSCs in 3D models, their gene expression signature, and their mutational and epigenetic profiles will undoubtedly lay a firm foundation to develop better therapeutic target specific compounds for this cell population. As a foundation in the development of personalized medicine, the high throughput screening of organoids isolated from Patient-Derived Cells (PDCs) is providing important information about the most appropriate drug strategies for the treatment of cancer [204–206]. The systematic study of CSCs' behavior and education of their niche through organoid culture, lineage tracing, single cell analysis, and bioinformatics will be instrumental to comprehend and target cancer development and persistence after therapy.

## 9. Conclusions 

Stemness is part of the normal repertoire of the genetic program of every cell and is very active during the first stages of development of any organism that reaches adulthood. CSCs use their stemness properties to perpetuate their lineage and survive stress and chemotherapy. The understanding of these mechanisms, first in normal adult SCs and then in CSCs in the context of their niche, is key to develop better therapeutic approaches. Stemness in cancer cannot be self-sustained. As in normal niches, it requires the configuration of complex cell-to-cell and matrix interactions generating the heterogeneity needed by the TME to maintain tumor progression. Comprehending the successive genetic and epigenetic changes in cancer cells to become a CSC or how CSCs thrive is currently allowing the development of applied knowledge based on targeting Cancer Stemness properties and reinforcing the present challenge to develop new preventive and healing strategies.

The TME as a heterogeneous mix of cells and noncellular components contributing to cancer progression should be considered an important element when novel cancer therapies are designed. Of particular relevance are MSCs that reside or are attracted to the TME and have the potential to foster cancer growth and generate immunoregulation using similar mechanisms as those observed in the normal SC niche [25]. MSCs and fibroblasts can change when faced with different types of cancer cells or conditioned mediums, generating CAFs [145]. New therapeutic approaches must be developed to target the interaction between cancer cells, MSCs, and fibroblasts as this process is linked to metastasis [167]. All TME stemness-related properties and genetic and epigenetic modifications in CSCs can be used as therapeutic targets, but they cannot be approached independently because compensatory mechanisms are activated that promote cancer survival [207, 208].

Finally, the understanding of the stemness properties shared by adult SCs and CSCs and their niches bring light to the fundamental question of how the TME organize and promote cancer progression and survival. New challenges include the tracking of the origin of CSCs and their progenitors as well as the quest to understand ways they educate other cells in the TME to help them grow and thrive in different types of cancer and in a wide range of patients. For these purposes, the use of high throughput assays for lineage tracing, single cell analysis, and organoid culture will find a place in novel research strategies. The combination of these techniques will hopefully elucidate the essential mechanisms for the maintenance of Cancer Stemness and will be instrumental in the design of more effective and personalized therapeutic approaches.

## Figures and Tables

**Table 1:** Comparison of traits of normal Stem Cells and Cancer Stem Cell biology.

Trait	Normal Stem Cells	Cancer Stem Cells
Self-renewal	High capacity [27, 28]	High capacity [10, 64, 70, 71]

Cell cycle duration	Long. Tissue-regulated generation of transit amplifying progenitors [28]	Redundant self-renewal pathways become activated. Pathological self-renewal balance over differentiation [72]

Genome repair abilities	Yes [73, 74]	Altered (constant generation of new mutations and epigenetic profiles to generate clones with strong adaption capacity to aggressive environments) [46, 73]. Hypoxia mediated cell cycle lengthening and DNA repair [75, 76]. Shorter cell cycle contribution [12]

Microenvironmental protection by niche from noxious agents	Yes [77]	Yes [11, 23]

Location at hierarchy	Basal compartment [27]	Basal compartment [72]

Transit amplifying compartment	Progenitor cells have short cycles to generate enough numbers of normal differentiated cells [47, 78]	It seems to be present as the basis for rapid growth of tumors. Progenitor cells have short cycles [10]

Plasticity	Can go back and forth between differentiation and dedifferentiation states [79]	Epithelial mesenchymal transition and self-renewal acquisition [10, 80, 81] Dedifferentiation and mutation accumulation in committed cells [22]

**Table 2:** Comparison of traits of normal Stem Cells and Cancer Stem Cell (CSCs) niches.

Trait	Normal Stem Cells	Cancer Stem Cells
Niche element: mesenchymal cells	Contribute with nutritional and stem-cell-fate factors [82]	Tumors educate the surrounding cells to provide nutrients, although highly resistant to lack of nutrients [83, 84]

Niche element: immune system cells	Modulate local environment for immune protection of SCs (immune suppression = immune-sanctuaries) [85]	Modulate local environment for immune protection of CSCs (immune suppression = immune-sanctuaries) [86]

Niche element: extracellular matrix	The matrix signals to SCs for fate regulation, promoting stemness and physiological maintenance [87]	Cancers cells produce large quantities of metalloproteinases, enzymes that degrade and remodel the ECM, thus promoting invasion and tumor angiogenesis [88]

Niche element: oxygen	Oxygen supply by blood vessels [89, 90]	Highly resistant to lack of oxygen [91], common in specific regions of tumors

Resistance to environmental stress	Highly resistant to cell death by noxious agents [92]	Highly resistant to lack of nutrients. Highly resistant to cell death by chemotherapy agents. Multidrug Resistance (MDR) pumps that extrude toxic compounds ATP-binding cassettes transporters (abc) [93] Quiescence activation through the ability to perceive stress [12, 24]
